# Principles of metadata organization at the ENCODE data coordination center

**DOI:** 10.1093/database/baw001

**Published:** 2016-03-15

**Authors:** Eurie L. Hong, Cricket A. Sloan, Esther T. Chan, Jean M. Davidson, Venkat S. Malladi, J. Seth Strattan, Benjamin C. Hitz, Idan Gabdank, Aditi K. Narayanan, Marcus Ho, Brian T. Lee, Laurence D. Rowe, Timothy R. Dreszer, Greg R. Roe, Nikhil R. Podduturi, Forrest Tanaka, Jason A. Hilton, J. Michael Cherry

**Affiliations:** 1 Department of Genetics, Stanford University School of Medicine Department of Genetics, Stanford, CA, USA; 2 Center for Biomolecular Science and Engineering Santa Cruz, University of California, Santa Cruz, CA, USA

## Abstract

The Encyclopedia of DNA Elements (ENCODE) Data Coordinating Center (DCC) is responsible for organizing, describing and providing access to the diverse data generated by the ENCODE project. The description of these data, known as metadata, includes the biological sample used as input, the protocols and assays performed on these samples, the data files generated from the results and the computational methods used to analyze the data. Here, we outline the principles and philosophy used to define the ENCODE metadata in order to create a metadata standard that can be applied to diverse assays and multiple genomic projects. In addition, we present how the data are validated and used by the ENCODE DCC in creating the ENCODE Portal (https://www.encodeproject.org/).

**Database URL:** www.encodeproject.org

## Introduction

The goal of the Encyclopedia of DNA Elements (ENCODE) project is to annotate functional regions in the human and mouse genomes. Functional regions include those that code protein-coding or non-coding RNA gene products as well as regions that could have a regulatory role (1, 2). To this end, the project has surveyed the landscape of the human genome using over 35 high-throughput experimental methods in > 250 different cell and tissue types, resulting in over 4000 experiments (1, 3). These datasets are submitted to a Data Coordinating Center (DCC), whose role is to describe, organize and provide access to these diverse datasets (4).

A description of these datasets, collectively known as metadata, encompasses, but is not limited to, the identification of the experimental method used to generate the data, the sex and age of the donor from whom a skin biopsy was taken, and the software used to align the sequencing reads to a reference genome. Defining and organizing the set of metadata that is relevant, informative and applicable to diverse experimental techniques is challenging. These challenges are not unique to the ENCODE DCC. Several major experimental consortia similar in scale to the ENCODE project exist, as well as public database projects that collect and distribute high-throughput genomic data. Analogous to the ENCODE project, the modENCODE project was begun in 2007 to identify functional elements in the model organisms *Caenorhabditis* *elegans* and *Drosophila* *melanogaster*. The modENCODE DCC faced similar challenges in trying to integrate diverse data types using a variety of experimental techniques (5). Other consortia, such as the Roadmap Epigenomics Mapping Centers, also have been tasked with defining the metadata (6). In addition, databases such as ArrayExpress at the EBI, GEO and SRA at the NCBI, Data Dryad (http://datadryad.org/) and FigShare (http://figshare.com/) serve as data repositories, accepting diverse data types from large consortia as well as from individual research laboratories (7–9).

The challenges of capturing metadata and organizing high-throughput genomic datasets are not unique to NIH-funded consortia and data repositories. Since many researchers submit their high-throughput data to data repositories and scientific data publications, tools and data management software, such as the Investigation Assay Study tools (ISA-tools) and Laboratory Information Management Systems (LIMSs), provide resources aimed to help laboratories organize their data for better compatibility with these data repositories (10). In addition, there have been multiple efforts to define a minimal set of metadata for genomic assays, including standards proposed by the Functional Genomics Data society (FGED; http://fged.org/projects/minseqe/) and the Global Alliance for Genomics and Health (GA4GH; https://github.com/ga4gh/schemas), to improve interoperability among data generated by diverse groups.

Here, we describe how metadata are organized at the ENCODE DCC and define the metadata standard that is used to describe the experimental assays and computational analyses generated by the ENCODE project. The metadata standard includes the principles driving the selection of metadata as well as how these metadata are validated and used by the DCC. Understanding the principles and data organization will help improve the accessibility of the ENCODE datasets as well as provide transparency to the data generation processes. This understanding will allow integration of the diverse data within the ENCODE consortium as well as integration with related assays from other large-scale consortium projects and individual labs.

## Metadata describing ENCODE assays

**Figure 1:**
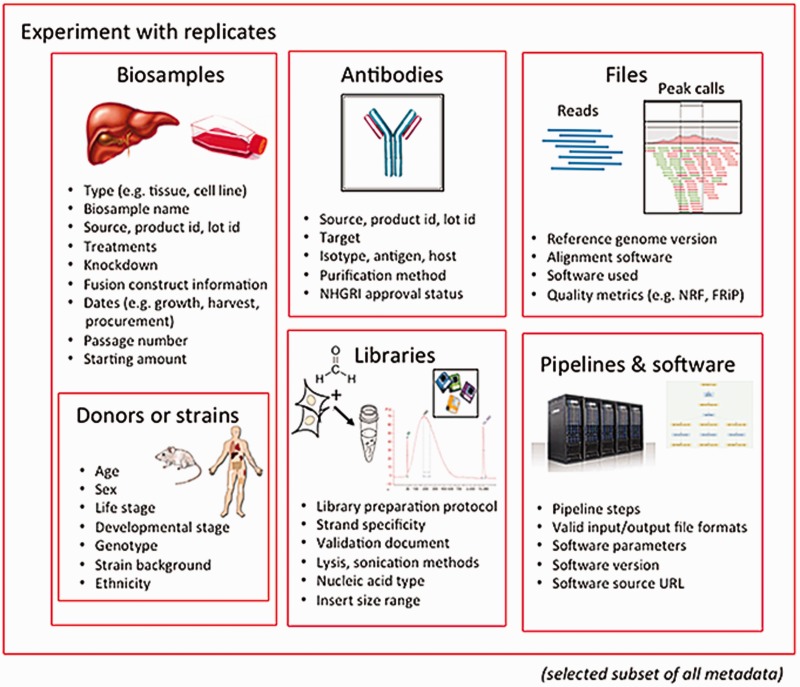
Major categories of metadata. The metadata captured for ENCODE can be grouped into the following major areas: biosamples and donors/strains (formerly ‘cell types’), libraries, antibodies, data files and pipelines and software. These categories are then grouped into an experiment with replicates. Only a subset of metadata is listed in the figure to provide an overview of the breadth and depth of metadata collected for an assay. The full set of metadata can be viewed at https://github.com/ENCODE-DCC/encoded/tree/master/src/encoded/schemas.

The categories of metadata currently being collected by the ENCODE DCC builds on the set collected during the previous phases of the project. During the earlier phases, a core set of metadata describing the assays, cell types and antibodies were submitted to the ENCODE DCC (11). The current metadata set expands the number of categories into the following major organizational units: biosamples, libraries, antibodies, experiments, data files and pipelines (Figure 1). Only a selected set of metadata are included below as examples, to give a sense of the breadth and depth of our approach.

The biological material used as input material for an experimental assay is called a biosample. This category of metadata is an expansion of the ‘cell types’ captured in previous phases of the ENCODE project (11). Biosample metadata includes non-identifying information about the donors (if the sample is from a human) and details of strain backgrounds (if the sample is from model organisms) (Figure 1). Metadata for the biosample includes the source of the material (such as a company name or a lab), how it was handled in the lab (such as number of passages or starting amounts) and any modifications to the biological material (such as the integration of a fusion gene or the application of a treatment).

The library refers to the nucleic acid material that is extracted from the biosample and contains details of the experimental methods used to prepare that nucleic acid for sequencing. Details of the specific population or sub- population of nucleic acid (e.g. DNA, rRNA, nuclear RNA, etc.) and how this material is prepared for sequencing libraries is captured as metadata.

The metadata recorded for antibodies include the source of the antibody, as well as the product number and the specific lot of the antibody if acquired commercially. Capturing the antibody lot id is critical because there is potential for lot-to-lot variation in the specificity and sensitivity of an antibody. Antibody metadata include characterizations of the antibody performed by the labs, which examines this specificity and sensitivity of an antibody, as defined by the ENCODE consortium (12).

The experiment refers to one or more replicates that are grouped together along with the raw data files and processed data files. Each replicate that is part of an experiment will be performed using the same experimental method or assay (e.g. ChIP-seq). A single replicate, which can be designated as a biological or technical replicate, is linked to a specific library and an antibody used in immunoprecipitation-based assay (e.g. ChIP-seq). Since the library is derived from the biosample, the details of the biosample are affiliated with the replicate through the library used.

A single experiment can include multiple files. These files include, but are not limited to, the raw data (typically sequence reads), the mapping of these sequence reads against the reference genome, and genomic features that are represented by these reads (often called ‘signals’ or ‘peaks’). Metadata pertaining to files include the file format and a short description, known as an output type, of the contents of the file.

In addition to capturing the format of the files generated (e.g. fastq, BAM, bigWig), metadata regarding quality control metrics, the software, the version of the software and pipelines used to generate the file are included as part of the pipeline-related set of metadata. The metadata for a given file also consist of other files that are connected through input and output relationships.

## Defining the metadata standard

The categories of metadata captured for the ENCODE experiments described in the previous section aim to provide a summary of the experimental and computational methods as well as provide enough information to facilitate the evaluation and interpretation of multiple experiments by the scientific community. The breadth and depth of metadata selected in each of the categories need to be able to uniquely identify experiments from each other. The following five principles guide the selection of the specific metadata in each category.

### Principle 1: Reflect experimental variables

The detailed metadata in each category are selected to reflect potential experimental variables when evaluating a set of similar assays. The key metadata may differ depending on the assays examined by the researcher so the set of metadata included for the ENCODE assays strives to be broadly applicable to multiple assays without sacrificing specificity for a single assay. To this end, extensive metadata describing the biosamples used in assays, preparation of the libraries and software used to generate the files are captured. Selected examples highlighting this principle are described.

The biosample metadata category includes donor information for human samples, such as the age and sex, or strain background information for model organisms, such as strain names and genotypes. Whether the biosample has been treated with a chemical or biological agent (such as tamoxifen or an infectious agent), contains a fusion protein (such as an eGFP tagged protein), or has been transfected with an RNAi to knock-down protein levels, also will be reflected in the biosample metadata category.

Preparation of libraries can dictate and influence which sets of data can be appropriately compared together, as well as influence the types of software that can be used to analyze the data. In particular, the processing of RNA-seq data differs on the length of the RNA population being selected, whether the RNA contains a 5′ 7-methylguanosine cap, and whether rRNA populations were removed prior to library prep. Although many of these details are not generally applicable to other assays, they are included in the ENCODE metadata set because they are essential in understanding the experimental variables for an RNA-seq assay.

The contents of a data file are strongly dependent on the software used to generate the file. Software with similar functions, such as an aligner, can produce different results. In addition, different parameters passed into a same software can result in different output. Therefore, file metadata category includes software information, consisting of version numbers, md5sum of the downloaded software and supporting documentation regarding parameters used.

### Principle 2: Help uniquely identify re-usable reagents

In addition to reflecting experimental variables, the metadata can provide sufficient information to differentiate between similar entities that are often used as reagents. The ability to distinguish between similar but not identical reagents is essential to ensure a specific biosample is used as input for different assays or the same lot of antibody is used for multiple ChIP-seq assays.

For example, tissues and primary cells can be differentiated by a difference between donors. Furthermore, identical biosamples from an individual donor, such as a blood draw, can be distinguished by recoding dates of collection for tissues and primary cell lines. Cell lines, whether immortalized or differentiated *in vitro*, can be distinguished as unique growths based on the date the batch was started. For antibodies, the lot number of the antibody is captured in addition to the vendor and product ID due to potential variation between lots.

### Principle 3: Encourage reproducibility and interpretation of the data

Another core principle of defining the ENCODE metadata standard is to include essential experimental and computational details that allow researchers to repeat the assays or analysis as well as provide insight and context to evaluate the quality of the experiment. For biosamples, the amount of the starting material used (in number of cells or weight) is included. In addition, metadata such as the source, including any product numbers and lot ids, will be recorded to allow other members of the scientific community to obtain similar biosamples. For libraries, this includes the methods used to lyse and prepare the nucleic acid for sequencing. For files, the versions of the software and the pipeline used are captured as well as the input files and reference files used to generate the output files of the analysis. Capturing this level of experimental details allows easier comparison between different assays, computational results and analyses.

### Principle 4: Represent data standards

The ENCODE Consortium has defined standards for a range of different aspects of the experimental and analysis process (https://www.encodeproject.org/data-standards/). The data standards include how assays should be performed as well as how data results should be analyzed. For example, there are standards agreed upon by the consortium for the consistent treatment of cells, including growth protocols and the number of passages. Standards describe how to evaluate the specificity and sensitivity of that antibody against the target have also been developed (12). In addition, standards for read depth and analysis methods are also agreed upon by the ENCODE Consortium. Since these are significant details about the assay as defined by the consortium, these specific metadata are included in the ENCODE metadata set. Growth protocols and other protocols for preparing the biosample and libraries are included as documents. Quality control metrics on read depth, uniquely mapping reads and replicate concordance values are captured as well.

### Principle 5: Facilitate searching and identification of experimental datasets

The metadata are used for searching data generated by the ENCODE Consortium at the ENCODE Portal (https://www.encodeproject.org/). In addition to previously mentioned metadata, other metadata were selected that can help improve searching and identification of the datasets of interest. For example, any set of files or assay can be associated with a citation in order to easily find data used in a given publication. In addition, the lab submitting the generated data is included to allow searching for data generated by a specific lab.

## Implementing the metadata principles

### Accessions

Within each of the categories of metadata, the uniquely identified experimental variables can help distinguish similar entities. In order to easily refer to these entities in data submission as well as in publications, ENCODE accessions are assigned to key metadata categories. Each ENCODE accession is stable and will be tracked once they are created.

**Figure 2:**
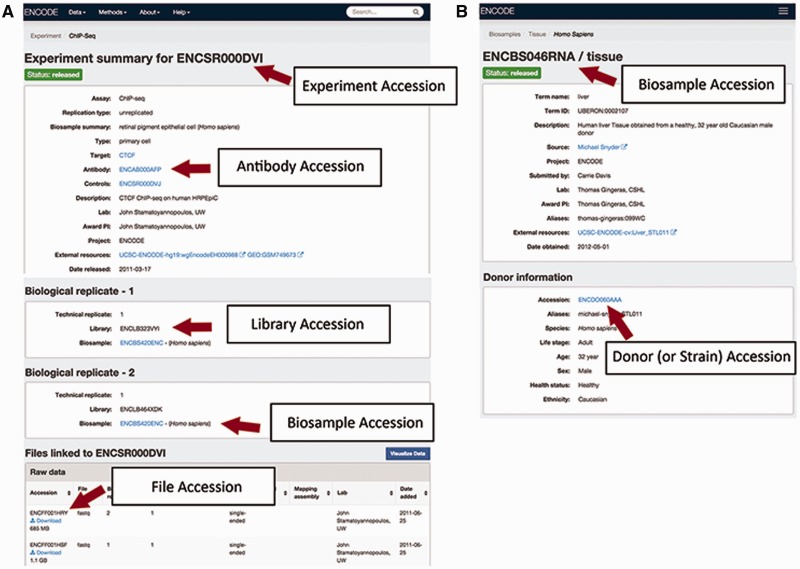
Accessions listed for an experiment on the ENCODE Portal. (A) An experiment page will contain accessions for the experiment referring to the full set of metadata describing how the assay was performed and the data generated by the assay, for the specific antibody lot used in that experiment, for the library that was generated, for the biosample that was used as input to the experiment, and for each data file generated by sequencing. (B) A biosample page will contain accessions for the biosample that was used as well as the unique donor (or strain) that provided the sample.

The accessions are in the format ENC[SR|BS |DO|AB| LB|FF] [0-9]{3} [A-Z]{3} where [SR|BS| DO|AB|LB |FF] refer to the metadata type given the accession (Figure 2). This allows for >17 million accessions to be generated by entity type. Accessions are given to the following types of metadata:

***An experiment***: An experiment accession refers to one or more replicates that are grouped together along with the raw data files and processed data files. Typically, each replicate will be performed using the same method, performed on the same kind of biosample and investigating the same target (see the ‘*Creating the ENCODE metadata data model*’ section for more details). A sample accession for an assay is ENCSR000DVI (Figure 2A).

***A biosample***: An accessioned biosample refers to a tube or sample of that biological material that is being used. For example, the following would all be given a biosample accession: (1) a batch of a cell line grown on a specific day, (2) the isolation of a primary cell culture on a specific day or (3) the dissection of a tissue sample on a specific day. An example of biosample accession is ENCBS046RNA (Figure 2B).

***A strain or donor***: Every strain background (for model organisms) and donor (for humans) is given a donor accession. This accession allows multiple samples obtained from a single donor to be grouped together. The donor information is listed within the biosample, for example in ENCBS046RNA (Figure 2B).

***An antibody lot***: Each unique combination of an antibody’s source, production number and lot is accessioned so that assays can refer specifically to that antibody. Each antibody lot is also associated with characterizations for its target in a species, for example in ENCAB934MDN (Figure 2A).

***A library***: A unique library that was sequenced is accessioned to ensure that the correct files are associated with the nucleic acid material that has been created from the biosample. The library accession and experimental details of how the library was constructed are displayed on the assay page, i.e. ENCSR000DVI (Figure 2A).

***A file***: Each data, analysis and reference file are accessioned. This accession is used as the file name, along with its file format as an extension. The file accession is associated with the contents of that file. When a new file is submitted to replace an existing file, the new file is given a new accession and related to the older file. Files are displayed at the bottom of an assay page, i.e. ENCSR000DVI (Figure 2A).

### Creating the ENCODE metadata data model

**Figure 3:**
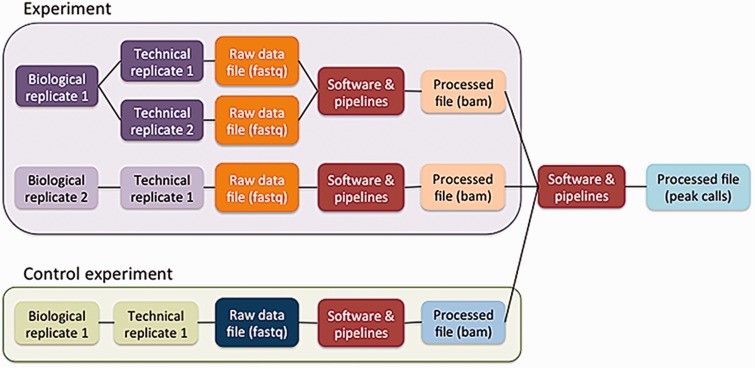
Schematic of the metadata model. The metadata model reflects how researchers perform laboratory and computational experiments. A single experiment can contain one or more replicates (see text). These replicates generate raw data files, which are then used in software and data processing pipelines to generate processed data files. Control experiments can be modeled similarly to experiments. Files from multiple experiments can be used as input for a single pipeline run.

The five principles are embodied in the ENCODE metadata model (https://github.com/ENCODE-DCC/encoded/tree/master/src/encoded/schemas or https://www.encodeproject.org/profiles/). The metadata are the details collected about the experiments and reagents, the metadata model is the definition of what can be collected, and the metadata data model is the computational structure used to store and organize the metadata model. The major categories of metadata are organized in a model that reflects how researchers perform the experimental and computational assays (Figure 3). Experiments in the metadata model can contain one or more replicates, representing the number of times an assay was performed in an attempt to demonstrate reproducibility. These repetitions can be classified as biological or technical replicates. Biological replicates, representing libraries made from distinct biosamples, can be further specified to indicate whether that biosample was derived from the same donor (isogenic) or from different donors (anisogenic). Technical replicates are linked to the same biosample. Although the ENCODE Consortium defines technical replicates as two different libraries of nucleic acid prepared from the same biosample, the metadata model can accommodate alternate definitions. Each replicate (either biological or technical) is linked to raw data files that are used in pipelines to generate additional processed files.

**Figure 4:**
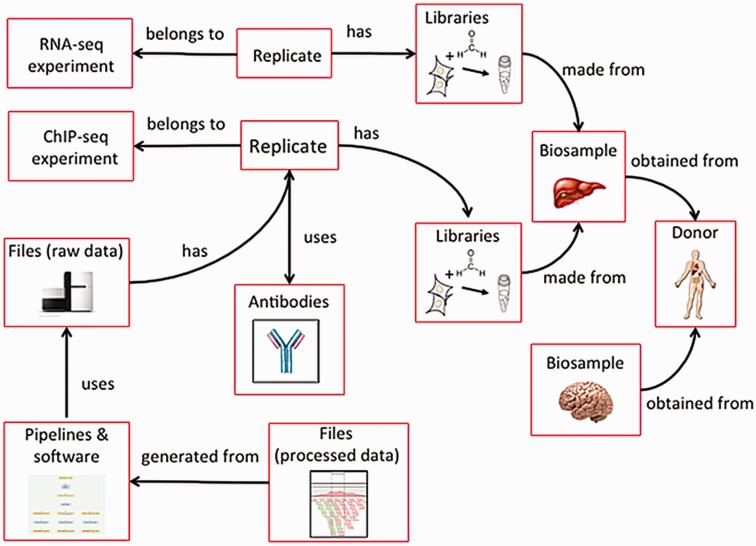
Categories in the metadata model are linked to each other. Categories of metadata are linked to each other and can be described by relationships between the categories. Each individual category can be referred to multiple times. For example, a liver and a brain can be obtained from the same donor. In addition, a single biosample, like the liver, can be used as input for multiple assays. Because each donor and biosample is accessioned, they can be referred to uniquely.

The ENCODE metadata data model is a hybrid relational-object-based data store in which the major categories of metadata are represented as one or more JSON objects implemented in JSON-SCHEMA and JSON-LD (Hitz *et* *al.*, in preparation). Because different aspects of experimental and computational assays are reused, each category of metadata that represents an experimental variable can be referred to multiple times (Figure 4). For example, a single donor can contribute multiple biosamples, such as a liver and brain, or multiple assays can use the same tissue.

The detailed metadata are captured as distinct structured fields or in protocol documents associated with the experiment. The decision as to whether the metadata are highlighted as a separate field is reflective of whether that specific metadata fulfills a set of the five criteria described above.

### Curation and validation of metadata

**Figure 5:**
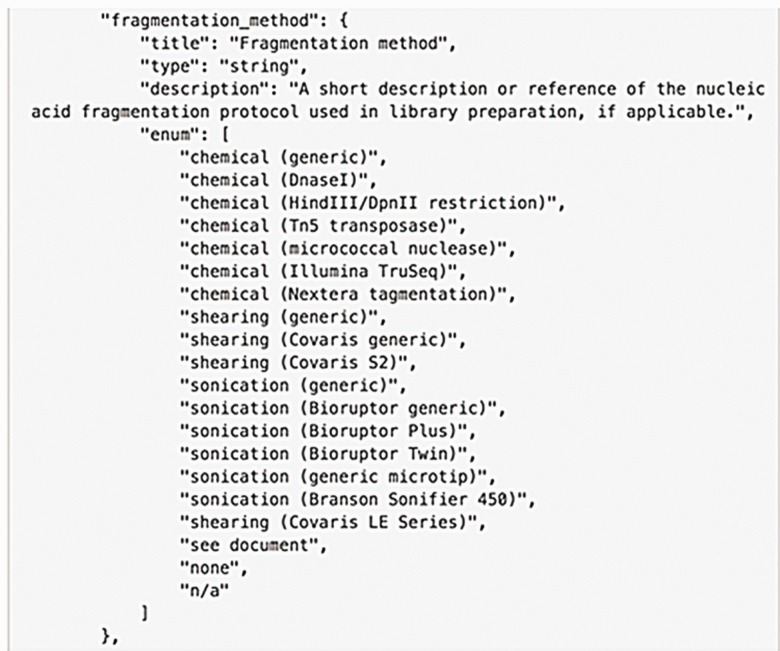
Example of an enumerated list in the schema. The metadata model is represented as a JSON object (this computational structure is the metadata data model) containing properties of specific metadata fields. An enumerated list is a list of allowed values for that property. It prevents typos or multiple spellings of a single item to maintain consistent data. Values added for this property are checked against the list when the data are added.

Use of controlled vocabularies and ontologies ensures consistent description of the metadata. The ENCODE DCC uses the appropriate ontologies, when available, that contain defined relationships used to capture the values for the metadata (13). These include the description of what the biosample is: UBERON for tissues, CL for primary cell types and EFO for immortalized cell lines (14–16). For treatments on the biosample, ChEBI terms will be used (17). OBI is used for capturing the assay name (18). The use of ontologies allows instant interoperability with other datasets that use the same ontologies (13). When no ontology is available, a controlled list of terms is provided in the schema as an enumerated list order to maintain rigorous consistency (Figure 5). The use of ontologies is more significant when multiple projects agree on their use as this allows interoperability between projects empowering greater use of their results.

**Figure 6:**
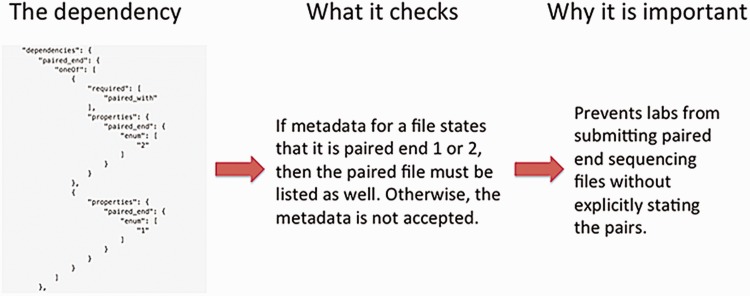
Metadata validation in the schema. The schema allows dependencies which allow conditions to be defined on which set of data should be submitted. In this example, the dependency states that the paired files from paired-end sequencing runs need to be explicitly defined. This prevents paired-end files from being separated from each other as the data are submitted.

**Figure 7:**
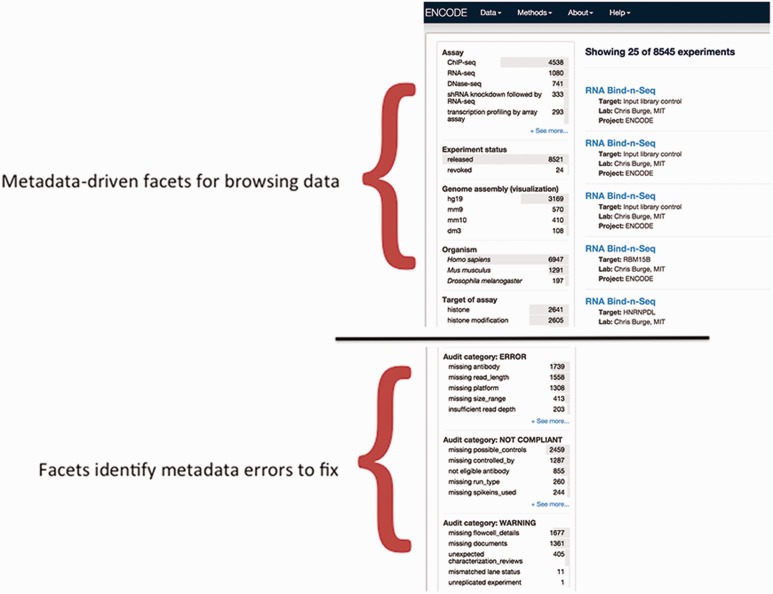
Validation of metadata using audits. The top half of the panel is a screenshot of the metadata-drive facets that can be used for browsing data. The bottom panel is a screenshot of a data audit that is visible to data submitters and the DCC. It includes a list of queries that are performed for inconsistent or incorrect metadata. These audits ensure that the metadata are accurate before data release.

The definition of a metadata standard and implementation of a data model are not sufficient to ensure a high- quality set of metadata. The metadata submitted by the ENCODE Consortium members are validated using features of JSON-SCHEMA upon submission to the DCC as well as through audits that identify inconsistencies in the data after submission. As previously mentioned, providing an enumerated list of accepted values for a metadata field in the JSON schema ensures that only that set of values are submitted to the DCC. Other features of JSON-SCHEMA implemented include dependencies which enforce the submission of metadata when related metadata are submitted. For example, if a fastq file is submitted, it is required to indicate whether the file contains data for a single-end or paired-end sequencing run. Subsequently, if the file contains information about the paired-ends, the paired file must be listed (Figure 6). In addition to validation of metadata upon submission, metadata are reviewed after submission using data audits to maintain data integrity (13). These data audits include checks that ensure that antibodies are submitted for ChIP-seq assays, controls are listed for assays when required for data analysis (such as ChIP-seq), and the spike-ins are used for RNA-seq assays (Figure 7).

### Metadata-driven searches

Metadata are essential to the understanding, interpretation and evaluation of genomic assays and analyses. In addition to providing these for the data generated by the ENCODE Consortium, the metadata drive the search tools at the ENCODE portal (https://www.encodeproject.org/) (Figure 7). Metadata are indexed in ElasticSearch (Hitz *et al.*, in preparation) and can be searched using keywords (4). In addition, selected metadata are used as filters that can be used in faceted browsing of the data (4). When ontologies are used for annotation, the relationships between the ontology terms are used to group related biosamples for easy searching (13). For example, a search of ‘skin’ will identify assays performed using ‘keratinocytes’ even though the text string ‘skin’ is not explicitly stated in the biosample name.

## Comparison with other models

As mentioned previously, other NIH-funded projects, scientific resources and researchers have developed systems for modeling and storing metadata for genomic assays. In developing the ENCODE metadata data model, the modENCODE metadata data model (5), ISA-TAB (19), GEO SOFT format (8) and Short Read Archive (SRA) XML (9) were reviewed for technical and practical feasibility in addition to meeting the five principles discussed above. They had to allow diverse assays to be stored in a common system, allow categories of metadata to refer to each other (e.g. many assays are performed on a single biosample or biosamples from the same donor, often involving multiple labs) (Figure 4), support the generation of web-based search tools and data audits and support the submission of a partial set of metadata (e.g. allow submission of data for one biological replicate before the second is ready). The ENCODE metadata data model was developed because none of the existing models could meet the defined requirements at the time our system was built. The efforts underway by the GA4GH initiative did not begin until after the ENCODE metadata data model was defined. The ENCODE metadata, however, can be exported in ISA-TAB, GEO, SRA-XML or GA4GH-defined formats.

FGED, developers of the widely adopted MIAME guidelines (20) and MAGE-TAB format (21), also developed a set of guidelines called MINSEQE to the Minimum information about a high-throughput nucleotide sequencing Experiment. The ENCODE metadata data model is MINSEQE compliant.

## Impact of the ENCODE metadata model

We have described the scope of the ENCODE metadata model, the principles behind the breadth and depth of the metadata included and essential features of its implementation. Because the ENCODE metadata model is structured around biologically and experimentally relevant units as opposed to consortium-specific activities, it has the potential to have broad impact by being flexible to accommodate sequencing-based assays not yet defined, being applied to data from other consortium-based projects, and promoting data provenance to support maximal data use.

The organization of metadata at the ENCODE DCC strives to represent how experimental assays are performed while maintaining a structure that is generic enough to accommodate additional sequencing-based assays that have not been adopted by the ENCODE consortium. Since the metadata model was initially designed, it has been able to accommodate assays types not used at the beginning of the project, such as ATAC-seq (22), with minimal modifications. In addition, the metadata model has been expanded to include metadata from other genomic projects. Metadata from modENCODE (23, 24) and the Epigenetics Roadmap project (25) have been re-curated to ENCODE metadata standards. The flexibility of the ENCODE metadata model to accommodate new assays and entirely new genomics projects suggests that it could be used for individual labs as well as other consortia.

And finally, the ENCODE metadata model allows transparency and reproducibility of the experimentally and computationally generated data. The categories of metadata and their implementation in a structured, machine-readable data model allow researchers to easily search and identify related experiments, understand how assays were performed and understand the provenance of data. Ultimately, the biggest impact will be the ability of the entire scientific community to accurately identify, re-use and integrate the ENCODE data.

As sequencing-based high-throughput assays become more widely adopted by individual labs and large consortia, the need to track experimental and computational methods used in an analysis becomes essential for reproducibility and data provenance. The metadata model described here, along with its organizing principles and key features for implementation, could be widely adopted as an open LIMS systems.

## References

[1] ENCODE Project Consortium (2012) An integrated encyclopedia of DNA elements in the human genome. Nature, 489, 57–74. 22955616 10.1038/nature11247 PMC3439153

[2] Yue F. Cheng Y. Breschi A, (2014) A comparative encyclopedia of DNA elements in the mouse genome. Nature, 515, 355–364. 25409824 10.1038/nature13992 PMC4266106

[3] Birney E. Stamatoyannopoulos J.A. ENCODE Project Consortium, (2007) Identification and analysis of functional elements in 1% of the human genome by the ENCODE pilot project. Nature, 447, 799–816. 17571346 10.1038/nature05874 PMC2212820

[4] Sloan C.A. Chan E.T. Davidson J.M, (2015) ENCODE data at the ENCODE portal. Nucleic Acids Res., 44, D726–D732. doi:10.1093/nar/gkv1160. 26527727 10.1093/nar/gkv1160 PMC4702836

[5] Washington N.L. Stinson E.O. Perry M.D, (2011) The modENCODE Data Coordination Center: lessons in harvesting comprehensive experimental details. Database, 2011, bar023. 21856757 10.1093/database/bar023 PMC3170170

[6] Bernstein B.E. Stamatoyannopoulos J.A. Costello J.F, (2010) The NIH roadmap epigenomics mapping consortium. Nat. Biotechnol., 28, 1045–1048. 20944595 10.1038/nbt1010-1045 PMC3607281

[7] Kolesnikov N. Hastings E. Keays M, (2015) ArrayExpress update—simplifying data submissions. Nucleic Acids Res., 43, D1113–D1116. 25361974 10.1093/nar/gku1057 PMC4383899

[8] Barrett T. Wilhite S.E. Ledoux P (2013) NCBI GEO: archive for functional genomics data sets—update. Nucleic Acids Res., 41, D991–D995. 23193258 10.1093/nar/gks1193 PMC3531084

[9] NCBI Resource Coordinators (2015) Database resources of the National Center for Biotechnology Information. Nucleic Acids Res., 43, D6–D17. 25398906 10.1093/nar/gku1130 PMC4383943

[10] Rocca-Serra P. Brandizi M. Maguire E, (2010) ISA software suite: supporting standards-compliant experimental annotation and enabling curation at the community level. Bioinformatics, 26, 2354–2356. 20679334 10.1093/bioinformatics/btq415 PMC2935443

[11] Rosenbloom K.R. Dreszer T.R. Long J.C, (2012) ENCODE whole-genome data in the UCSC Genome Browser: update 2012. Nucleic Acids Res., 40, D912–D917. 22075998 10.1093/nar/gkr1012 PMC3245183

[12] Landt S.G. Marinov G.K. Kundaje A, (2012) ChIP-seq guidelines and practices of the ENCODE and modENCODE consortia. Genome Res., 22, 1813–1831. 22955991 10.1101/gr.136184.111 PMC3431496

[13] Malladi V.S. Erickson D.T. Podduturi N.R, (2015) Ontology application and use at the ENCODE DCC. Database, 2015. doi:10.1093/nar/bav010. 10.1093/database/bav010 PMC4360730 25776021

[14] Mungall C.J. Torniai C. Gkoutos G.V, (2012) Uberon, an integrative multi-species anatomy ontology. Genome Biol., 13, R5. 22293552 10.1186/gb-2012-13-1-r5 PMC3334586

[15] Bard J. Rhee S.Y. Ashburner M. (2005) An ontology for cell types. Genome Biol., 6, R21. 15693950 10.1186/gb-2005-6-2-r21 PMC551541

[16] Malone J. Holloway E. Adamusiak T, (2010) Modeling sample variables with an experimental factor ontology. Bioinformatics, 26, 1112–1118. 20200009 10.1093/bioinformatics/btq099 PMC2853691

[17] Hastings J. de Matos P. Dekker A, (2013) The ChEBI reference database and ontology for biologically relevant chemistry: enhancements for 2013. Nucleic Acids Res., 41, D456–D463. 23180789 10.1093/nar/gks1146 PMC3531142

[18] Brinkman R.R. Courtot M. Derom D, (2010) Modeling biomedical experimental processes with OBI. J. Biomed. Semantics, 1(Suppl 1), S7. 20626927 10.1186/2041-1480-1-S1-S7 PMC2903726

[19] Sansone S.A. Rocca-Serra P. Field D, (2012) Toward interoperable bioscience data. Nat. Genet., 44, 121–126. 22281772 10.1038/ng.1054 PMC3428019

[20] Brazma A. Hingamp P. Quackenbush J, (2001) Minimum information about a microarray experiment (MIAME)-toward standards for microarray data. Nat. Genet., 29, 365–371. 11726920 10.1038/ng1201-365

[21] Rayner T.F. Rocca-Serra P. Spellman P.T, (2006) A simple spreadsheet-based, MIAME-supportive format for microarray data: MAGE-TAB. BMC Bioinformatics, 7, 489. 17087822 10.1186/1471-2105-7-489 PMC1687205

[22] Buenrostro J.D. Giresi P.G. Zaba L.C (2013) Transposition of native chromatin for fast and sensitive epigenomic profiling of open chromatin, DNA-binding proteins and nucleosome position. Nat. Methods, 10, 1213–1218. 24097267 10.1038/nmeth.2688 PMC3959825

[23] Gerstein M.B. Lu Z.J. Van Nostrand E.L, (2010) Integrative analysis of the *Caenorhabditis elegans* genome by the modENCODE project. Science, 330, 1775–1787. 21177976 10.1126/science.1196914 PMC3142569

[24] Roy S. Ernst J. modENCODE Consortium, (2010) Identification of functional elements and regulatory circuits by Drosophila modENCODE. Science, 330, 1787–1797. 21177974 10.1126/science.1198374 PMC3192495

[25] Kundaje A. Meuleman W., Roadmap Epigenomics Consortium, (2015) Integrative analysis of 111 reference human epigenomes. Nature, 518, 317–330. 25693563 10.1038/nature14248 PMC4530010

